# Effect of Heat Stress on Bovine Mammary Cellular Metabolites and Gene Transcription Related to Amino Acid Metabolism, Amino Acid Transportation and Mammalian Target of Rapamycin (mTOR) Signaling

**DOI:** 10.3390/ani11113153

**Published:** 2021-11-04

**Authors:** Lin Fu, Li Zhang, Li Liu, Heng Yang, Peng Zhou, Fan Song, Guozhong Dong, Juncai Chen, Gaofu Wang, Xianwen Dong

**Affiliations:** 1 Chongqing Academy of Animal Sciences, Chongqing 402460, China; lyfl1990@163.com (L.F.); zhangli03094@163.com (L.Z.); cqzp2006@163.com (P.Z.); songfanjx@163.com (F.S.); 2 Faculty of Pharmaceutical Engineering, Chongqing Chemical Industry Vocational College, Chongqing 401228, China; Lycqhgzyxy@163.com; 3 College of Veterinary Medicine, Southwest University, Chongqing 402460, China; yh20183007@swu.edu.cn; 4 College of Animal Science and Technology, Southwest University, Chongqing 400716, China; gzdong@swu.edu.cn (G.D.); juncai.chen@hotmail.com (J.C.)

**Keywords:** heat stress, metabolomics, amino acid metabolism, milk protein synthesis, MAC-T cell

## Abstract

**Simple Summary:**

This study mainly employed metabolomics technology to determine changes of intracellular metabolite concentrations related to milk protein synthesis induced by heat stress (HS) in bovine mammary epithelial cells. HS was associated with significant differences in intracellular amino acid metabolism resulting in an increase in the intracellular amino acid concentrations. Moreover, HS promoted amino acid transportation and the activity of the mammalian target of rapamycin (mTOR) signaling pathway, which plays an important role as a central regulator of cell metabolism, growth, proliferation and survival. Greater expression of the alpha-S2-casein gene (*CSN1S2*) was also observed during HS. Overall, our study indicated that bovine mammary epithelial cells may have the ability to resist HS damage and continue milk protein synthesis partly through enhanced intracellular amino acid absorption and metabolism and by activating the mTOR signaling pathway during HS.

**Abstract:**

Heat stress (HS) is one of the most serious factors to negatively affect the lactation performance of dairy cows. Bovine mammary epithelial cells are important for lactation. It was demonstrated that HS decreases the lactation performance of dairy cows, partly through altering gene expression within bovine mammary epithelial tissue. However, the cellular metabolism mechanisms under HS remains largely unknown. The objective of this study was to determine whether HS induced changes in intracellular metabolites and gene transcription related to amino acid metabolism, amino acid transportation and the mTOR signaling pathway. Immortalized bovine mammary epithelial cell lines (MAC-T cells, *n* = 5 replicates/treatment) were incubated for 12 h at 37 °C (Control group) and 42 °C (HS group). Relative to the control group, HS led to a greater mRNA expression of heat shock protein genes *HSF1, HSPB8, HSPA5, HSP90AB1* and *HSPA1A*. Compared with the control group, metabolomics using liquid chromatography tandem–mass spectrometry identified 417 differential metabolites with *p* < 0.05 and a variable importance in projection (VIP) score >1.0 in the HS group. HS resulted in significant changes to the intracellular amino acid metabolism of glutathione, phenylalanine, tyrosine, tryptophan, valine, leucine, isoleucine, arginine, proline, cysteine, methionine, alanine, aspartate and glutamate. HS led to a greater mRNA expression of the amino acid transporter genes *SLC43A1*, *SLC38A9*, *SLC36A1*, and *SLC3A2* but a lower mRNA expression of *SLC7A5* and *SLC38A2*. Additionally, HS influenced the expression of genes associated with the mTOR signaling pathway and significantly upregulated the mRNA expression of mTOR, *AKT*, *RHEB*, *eIF4E* and *eEF2K* but decreased the mRNA expression of *TSC1, TSC2* and *eEF2* relative to the control group. Compared with the control group, HS also led to greater mRNA expression of the *CSN1S2* gene. Overall, our study indicates that bovine mammary epithelial cells may have the ability to resist HS damage and continue milk protein synthesis partly through enhanced intracellular amino acid absorption and metabolism and by activating the mTOR signaling pathway during HS.

## 1. Introduction

HS negatively impacts animal health and production parameters [1]. For dairy cows, HS results in a decrease in milk production and milk content synthesis through an increase in the additional heat load on a cow’s body [2]. Although environmental cooling systems could partly ameliorate HS conditions on dairy cow herds, the economic loss is still over USD 1.2 billion annually [3]. Given the trend of rising global temperature, there will be an estimated 1.5 °C increase between 2030 and 2052 [4]. Therefore, it is essential to further study the intracellular effects induced by HS on dairy cows to improve and inform appropriate management strategies.

Traditional studies found that HS impaired milk yield through a decrease in food intake [5]. However, more recently, studies performed using pair-fed thermal neutral cows have demonstrated that the decrease in dry matter intake (DMI) only accounted for approximately 30–50% of the milk yield reduction over the milk production period, indicating that other factors negatively affect milk yield [6]. Bovine mammary epithelial cells (BMECs) have demonstrated HS-sensitive characteristics, resulting in the reduction of milk synthesis and secretion. Prior research in vitro and in vivo indicated that the number and activity of BMECs significantly decreased with HS stimulation [7,8]. Moreover, HS upregulated the mRNA expression of apoptosis and heat shock genes but significantly downregulated the mRNA expression of genes involved in cell integrity and biosynthesis, especially the amino acid transporter and casein related to milk protein synthesis [9,10]. These studies suggest that HS could change the BMEC function potential through altered gene expression. The change in BMEC intracellular metabolites during HS has not been characterized to date.

Metabolomics is an emerging and powerful approach for elucidating the change of metabolites or chemical compounds generated by low-molecular-weight cells and tissues using gas chromatography–mass spectrometry (GC–MS), liquid chromatography tandem–mass spectrometry (LC–MS) and nuclear magnetic resonance (NMR) [11,12]. Recently, metabolomics has been used to investigate metabolic alterations in rumen fluid, serum, milk, urine and the mammary gland in dairy cows, with the aim of identifying diagnostic biomarkers and special metabolic pathways related to nutrition treatment, hepatic steatosis and mastitis, in order to improve productivity [13,14,15,16]. Our previous research confirmed that amino acid ratios (ratio of lysine to methionine and ratio of branched amino acids) could influence milk protein synthesis via the regulation of intracellular metabolite regulation in BMECs [17,18]. To our knowledge, metabolomics technology has been previously utilized to study the effect of HS on metabolites in the serum, rumen fluid and milk of dairy cows but not on the intracellular metabolites of BMECs.

Therefore, our hypothesis was that BMECs might have the ability to adapt to HS and maintain milk yield and milk components partly through the regulation of intracellular metabolism. To address this hypothesis, the immortalized BMEC line (MAC-T) was cultured with different temperatures (37 and 42 °C) and LC-MS metabolomics technology and gene transcription were used to analyze the biological response in HS.

## 2. Materials and Methods

### 2.1. Cell Culture and Treatments

The immortalized BMEC line (MAC-T) was chosen as the model. Cell culture was similarly performed base on our previous protocol [17]. In brief, MAC-T cells were recovered in 75 cm^2^ flasks with the condition of 37 °C and 5% CO_2_ in an incubator. Then cell culture was performed until the number of cells was sufficient for the experiment. The basal medium was prepared using DMEM/F12 (Thermo Fisher Scientific, South Logan, UT, USA) with 10% fetal bovine serum (FBS) and 100 U/mL penicillin/streptomycin (Thermo Fisher Scientific, South Logan, UT, USA). The experiment medium was the same as the basal medium except for FBS being replaced with bovine serum albumin (BSA). For HS research, MAC-T cells were cultured at 37 °C (control group, CON) and 42 °C (heat stress group, HS) for 12 h with five duplicates, respectively [19]. For the intracellular metabolomics analysis, cells were incubated in a culture dish (1.8 × 10^6^ cells, 100 mm) until they reached 80% to 90% confluence. Then cells were incubated at different temperatures (37 and 42 °C [20,21]) after serum-free starvation overnight. Finally, MAC-T cells were collected into 15 mL tubes and stored at −80 °C until metabolomics analyses were performed. To determine the effect of HS on gene expression in MAC-T cells, the treatments and cell culture procedure were as the same as that for the metabolomics analyses except that six-well plates were used to in place of culture dishes.

### 2.2. RNA Extraction and Real-Time PCR Analysis

The RNA extraction and real-time PCR (RT-PCR) with five replicates were performed base on our previous reports [17]. Briefly, total RNA was extracted from MAC-T cells using TRIzol reagent (Invitrogen, Carlsbad, CA, USA) and RNA quality of each sample was quantified through NanoDrop 1000 ND-2000 spectrophotometer (Thermo Scientific, Waltham, MA, USA). The cDNA synthesis was performed using the PrimeScript RT reagent Kit with gDNA Eraser (Takara Biotechnology, Dalian, China) according to the manufacturer’s instructions. The RT-PCR was performed according to the manufacturer’s instructions of SYBR Premix Ex Taq (Takara Biotechnology, Dalian, China). The cDNA was diluted to 50 ng with RNase-free water, 2 μL of diluted cDNA was combined in the 20 μL reaction mixture. The 20 μL system also contained 10 μL of 2 × SYBR Premix Ex Taq (Takara Biotechnology, Dalian, China), 0.4 μL each of 10 μM forward and reverse primers, 0.4 μL of 50 × ROX Reference Dye II and 4.8 μL of RNase-free water. All RT-PCR analyses were performed in QuantStudio 6 Flex System (Applied Biosystems, Foster City, CA, USA) with the following program: 95 °C for 30 s, 40 cycles at 95 °C for 5 s, and 60 °C for 34 s. Primer design and verification was performed based on the protocols reported in Loor laboratory [22]. The primer information of target genes, that is, heat shock protein genes, casein genes, amino acid transporter genes and mTOR signaling pathway genes, is shown in Supplemental Table S1 [6,18]. Three housekeeping genes (*GADPH*, *UXT* and *RPS9*) were used as the internal control against which target gene expression was normalized. The mRNA expression levels of the target genes in arbitrary units were calculated from the value of the threshold cycle (Ct) of RT-PCR compared to that of the internal control performing by the comparative cycle threshold (2^−∆∆Ct^) method (∆Ct = Ct_gene of target_ − Ct_internal control_, ∆∆Ct = ∆Ct_gene of HS group_ − Ct_gene of control group_) [23]. The results were presented as means ± standard deviation.

### 2.3. LC-MS Analysis

The comparative metabolomics analysis was performed based on the LC–MS. For metabolite extraction, the solvent was added to 50 mg of sample (acetonitrile–methanol-water, 2:2:1, containing internal standard, 1 mL), followed by vortexing for 30 s, and homogenized at 45 Hz for 4 min, then incubated at −20 °C for 1 h and centrifuged at 12,000 rpm for 15 min. Finally, the supernatant was extracted and stored at −80 °C.

LC-MS analysis was performed using a UHPLC system (1290, Agilent Technologies, Santa Clara, CA, USA) with a UPLC HSS T3 column (2.1 mm × 100 mm, 1.8 μm) coupled to Q Exactive (Orbitrap MS, Thermo). The column temperature was maintained at 45 °C, and the injection volume was 2 μL. The MS was set up in positive (POS) and negative (NEG) ionization mode. Mobile phase A was composed of 0.1% formic acid in water for POS and 5 mmol/L ammonium acetate in water for NEG, and the mobile phase B was acetonitrile. The flow rate was 0.5 mL/min with a gradient elution as follows: 0 min, 1% acetonitrile; 1 min, 1% acetonitrile; 8 min, 99% acetonitrile; 10 min, 99% acetonitrile; 10.1 min, 1% acetonitrile; 12 min, 1% acetonitrile. MS/MS spectra were received by using the QE mass spectrometer on an information-dependent basis (IDA). OSI-SMMS (version 1.0, Dalian Chem Data Solution Information Technology Co. Ltd., Dalian, China) was used for peak annotation after data processing.

### 2.4. Data Analysis

All the RT-PCR expression data for each gene were log_2_ transformed to obtain a normal distribution before statistical analyses. All statistical analyses were performed by the MIXED model in SAS (version 9.3; SAS Institute Inc., Cary, NC, USA) with temperature as a fixed effect and individual cell culture well or dish as the random effect. Means of treatment were generated by the LSMEANS option and separated using the PDIFF option with significance *p* < 0.05. Data were presented as mean ± standard error of mean. For metabolomics analysis, praetor scaling was applied to reduce noise and artifacts in the models. Data were analyzed by principle component analysis (PCA) to monitor the reproducibility of the instrument and (orthogonal) partial least-squares-discriminant analysis (OPLS-DA) were applied to establish the differential analysis of metabolic characteristics. Models with parameters R^2^Y and Q^2^ greater than 0.5 were regarded as having prominent predictive ability. The VIP score of the OPLS-DA model was applied to rank the metabolites that best distinguished between the two groups (VIP > 1). In addition, independent t-tests (*p* < 0.05) were also used to determine the significantly different results that the candidate biomarkers obtained from OPLS-DA of the control and HS group [24,25,26]. Metabolite abundances were calculated through the PMR and Metabolites database.

## 3. Results

### 3.1. Heat Shock Response of MAC-T Cells

**Figure 1:**
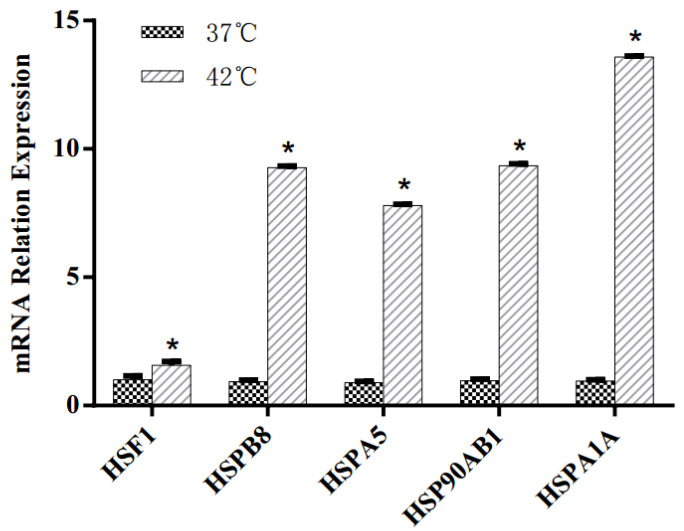
Effects of heat stress on mRNA expression of heat shock response genes in MAC-T bovine mammary epithelial cells. Asterisks indicated significant differences between different groups: * *p* < 0.05.

Heat shock response was triggered by high-temperature treatment at 42 °C in MAC-T cells. The effects of HS on mRNA expression of heat shock response genes are shown in Figure 1. Compared with control group incubated at 37 °C, HS significantly upregulated the gene expression of heat shock factor 1 (*HSF1*), heat shock protein beta-8 (*HSPB8*), heat shock protein 5 (*HSPA5*), heat shock protein 90 kDa alpha, class B member 1 (*Hsp90AB1*) and heat shock 70 kDa protein 1A (*HspA1A*) in this study (*p* < 0.05).

### 3.2. Intracellular Metabolism of MAC-T Cells

Metabolomics analysis showed that a total of 12,176 and 9737 compounds were detected by the POS mode and NEG mode, respectively. From these compounds, 7176 and 6020 metabolites were identified and quantified, respectively. More detailed information is presented in Supplemental Table S2. For the identification and analysis of differential metabolites between different groups, the metabolic molecules detected in the POS and NEG ionization modes were combined and the duplicates were removed by using MS2 spectra identification analysis results.

**Figure 2:**
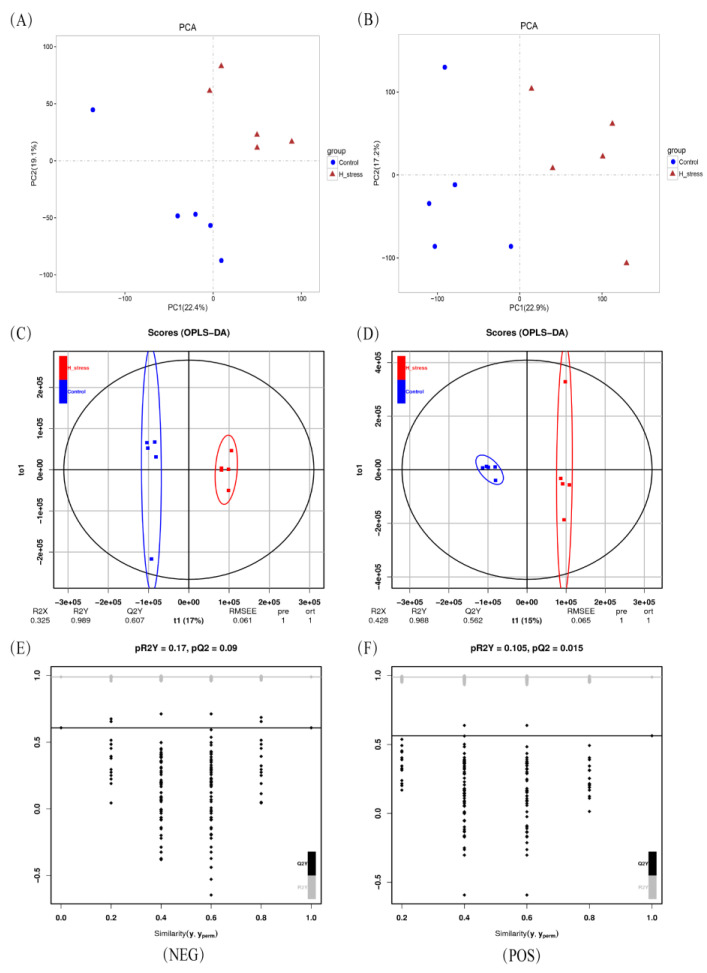
Metabolomic analysis of PCA score map, OPLS-DA score plot and permutation test of OPLS-DA. Control group (incubation at 37 °C, *n* = 5) and treatment group (incubation at 42 °C, *n* = 5). Multivariate analysis of (**A**,**C**,**E**) was performed base on negative ion (NEG) mode, while the multivariate analysis (fixed effect = temperature, random effect = culture plate) of (**B**,**D**,**F**) was performed based on positive ion mode (POS). PCA = principal component analysis, the blue represent control, and the red represent heat stress. OPLS-DA = orthogonal partial least squares discriminant analysis, the blue represents control, and the red represents heat stress.

The multivariate analyses of the metabolic profiles revealed unique clusters between the control group and HS group in both POS and NEG ionization modes (Figure 2A–D). In the OPLS-DA model, the parameter R^2^Y was greater than 0.988, and the Q^2^ values were greater than 0.56, suggesting good reliability and predictive ability of the model used in this study. The 200 permutation test of the OPLS-DA model performed to avoid overfitting of the parameters pR^2^Y (0.17) and pQ^2^ (0.09) yielded a result less than 1.0, indicating that the model was appropriate for further analysis with good robustness and validity (Figure 2E,F).

**Figure 3:**
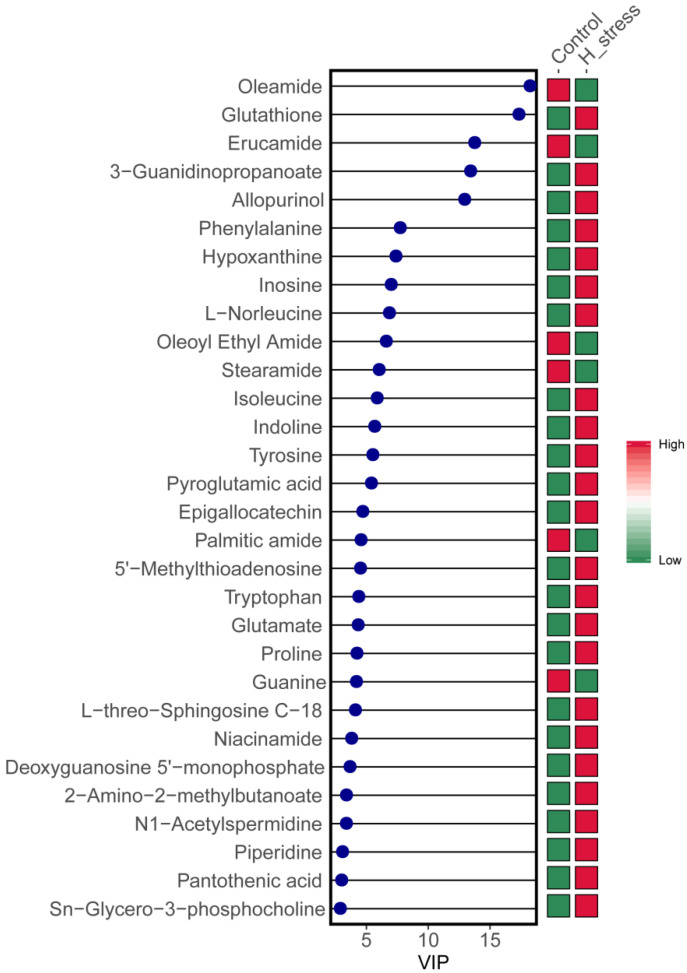
Variable importance in projection (VIP) score analysis plot of intracellular important metabolite in immortalized bovine mammary cell lines (MAC-T). The top 30 important metabolites were arranged from top to bottom according to intracellular concentration. The red box represents a high concentration of the molecule and the green box represents low concentration.

A total of 417 differential metabolites with *p* < 0.05 and VIP > 1.0 were obtained based on PLS-DA analysis, with 219 increased and 198 decreased in the HS group relative to the control group. The 30 metabolites shown in Figure 3 represent at least 8 metabolic pathways: glutathione metabolism; phenylalanine, tyrosine and tryptophan metabolism; valine, leucine and isoleucine metabolism; alanine, aspartate and glutamate metabolism; arginine and proline metabolism; lipid metabolism; nucleotide metabolism; metabolism of cofactors and vitamins. Additionally, 15 metabolites (glutathione, phenylalanine, L-norleucine, isoleucine, tyrosine, pyroglutamic acid, 5′-methylthioadenosine, tryptophan, glutamate, proline, L-threo-sphingosine C-18, 2-amino-2-methylbutanoate, N1-acetylspermidine, pantothenic acid and sn-glycero-3-phosphocholine) related to amino acid metabolism were observed [27,28,29], indicating that HS exerted a dramatic effect on intracellular amino acid metabolism in MAC-T.

**Figure 4:**
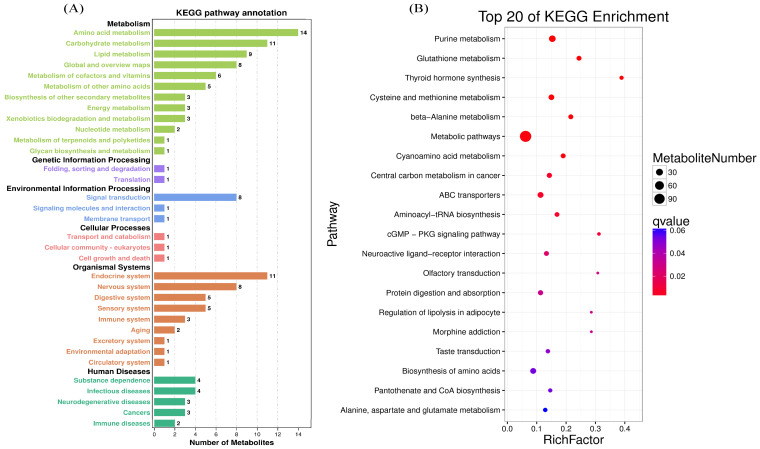
KEGG-pathway-enrichment analysis. Significant differences between different groups: * *p* < 0.05. (**A**) The KEGG pathway primary classification. The horizontal axis represents the number of differential secondary-level pathways, and the vertical axis represents the pathway. Different colors represent different secondary-level pathways classified by the system. (**B**) The three-level classification KEGG enrichment of top 20 pathways according to the q-value Different colors represent different adjusted q-values, from blue to red, indicating that the adjusted *p*-values are increasing from large to small, and the degree of enrichment is becoming more and more significant. The size of the dot represents the number of genes enriched in this pathway.

For more in-depth biological function information, KEGG pathway annotation analysis was performed according to significant (*p* < 0.05, VIP > 1.0) differential metabolites with the assistance of KEGG database. A total of 417 differential metabolites were classified into metabolism, organismal systems, human diseases, genetic information processing and environmental information processing (Figure 4A). As expected, amino acid metabolism pathways had the highest number of significant differential metabolites in response to HS. Furthermore, the metabolism-enrichment pathways with *p* < 0.05 are shown in Figure 4B, grouping into glucose, lipid, nucleotide, amino acid, vitamin and other material and energy metabolism pathways. Pathways of glutathione metabolism, cysteine and methionine, beta-alanine metabolism, ABC transporters, aminoacyl-tRNA biosynthesis, protein digestion and absorption, biosynthesis of amino acids and alanine, aspartate and glutamate metabolism are directly related to amino acid metabolism (Figure 4B).

### 3.3. Milk Protein Synthesis Regulation of MAC-T Cells

**Figure 5:**
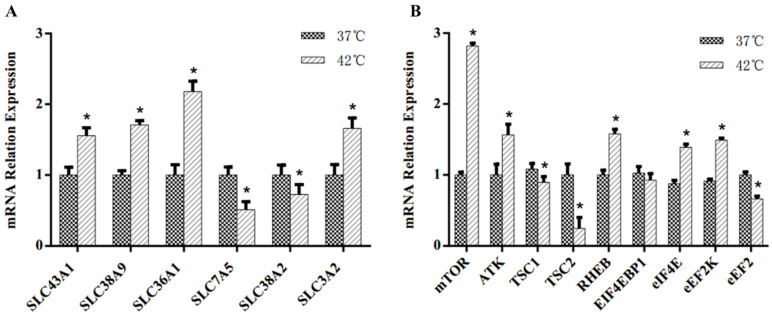
Effects of heat stress on mRNA expression of amino acid transporter and key regulator genes at mTOR signaling pathway in MAC-T bovine mammary epithelial cell line cultured at 42 °C. (**A**) The mRNA expression of amino acid transporter genes. (**B**) The mRNA relation expression of key regulator genes at mTOR signaling pathway. Asterisks indicate significant differences between different groups: * *p* < 0.05.

The effects of HS on the mRNA abundance of amino acid transporter and key regulator genes at the mTOR signaling pathway are shown in Figure 5. Compared with the control group, the HS group with a cell culture temperature of 42 °C had greater (*p* < 0.05) mRNA abundance of the amino acid transporter genes large neutral amino acid transporter small subunit 3 (*SLC43A1*), sodium-coupled neutral amino acid transporter 9 (*SLC38A9*), proton-coupled amino acid transporter 1 (*SLC36A1*) and 4F2 cell-surface antigen heavy chain (*SLC3A2*), but lower (*p* < 0.05) mRNA abundance of large neutral amino acids transporter small subunit 1 (SLC7A5) and sodium-coupled neutral amino acid transporter 2 (*SLC38A2*) was observed. Compared with the control group, the HS group had greater (*p* < 0.05) mRNA abundance of mTOR, serine/threonine-protein kinase (*AKT*), GTP-binding protein Rheb (*RHEB*), eukaryotic translation initiation factor 4E (*eIF4E*) and eukaryotic elongation factor 2 kinase (*eEF2K*) in the mTOR signaling pathway. However, lower (*p* < 0.05) mRNA abundance of tuberous sclerosis complex 1 (*TSC1*), tuberous sclerosis complex 2 (*TSC2*) and elongation factor 2 (*eEF2*) were observed. There was no significant difference in the mRNA expression of eukaryotic translation initiation factor 4E binding protein 1 (EIF4EBP1) in both the control group and the treatment group.

### 3.4. Casein Synthesis of MAC-T Cells

**Figure 6:**
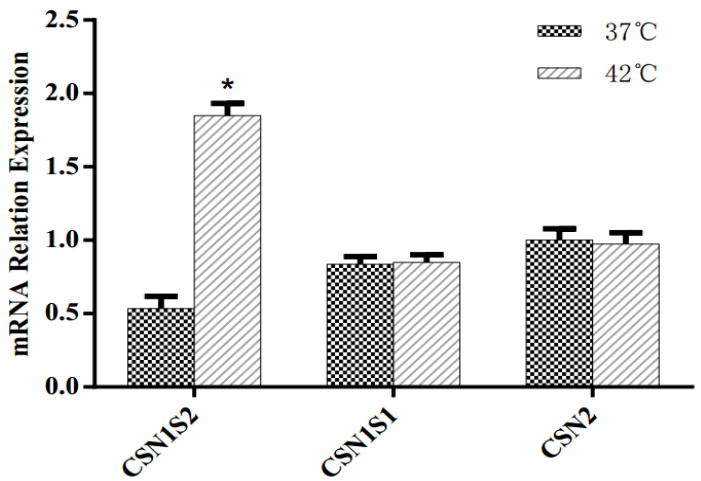
Effects of heat stress on mRNA expression of casein gene in immortalized bovine mammary cell lines (MAC-T) cultured at 42 °C. Asterisks indicate significant differences between different groups: * *p* < 0.05.

The effects of HS on the mRNA expression of casein genes are reported in Figure 6. In response to HS, the MAC-T cells displayed significantly increased expression of *CSN1S2*. No significant difference in the mRNA expression of *CSN1S1* and *CSN2* genes was observed with incubation at 42 °C relative to 37 °C.

## 4. Discussion

### 4.1. Heat Stress Model Construction

Heat shock proteins (HSPs) that function to protect cells from HS by repairing protein damage and maintaining normal growth [10] were confirmed in the 42 °C treatment group but not in the 37 °C control group. HS induces greater mRNA expression of *HSPB8*, *HSPA5*, *Hsp90AB1* and *HspA1A* in BMECs [10]. HS also increased the mRNA expression of *HSF1*, which was confirmed by the ability to block apoptosis during HS due to its regulatory function of genes encoding molecular chaperones [19]. As expected, HS significantly increased the mRNA expression of *HSF1*, *HSPB8*, *HSPA5*, *HSP90AB1* and *HSPA1A* in this study, which is in accordance with previous studies that showed HS led to the induction of HSP genes [30,31]. These results suggest that an appropriate HS MAC-T BMEC model was achieved by culturing at 42 °C for 12 h.

### 4.2. Metabolomics

This study has demonstrated that HS causes the metabolite profiles involved in amino acid metabolism to change in BMECs. The present study also demonstrated changes in intracellular metabolites and gene transcription related to amino acid metabolism, amino acid transportation and mTOR signaling in BMECs. Previously, it was demonstrated that the metabolite concentration involved in amino acid metabolism, lipolysis metabolism and glycolysis metabolism were with significantly changed in the plasma and milk of dairy cows exposed to HS [31,32]. Moreover, the significant change in amino acid concentration induced by HS was also observed in the serum of dairy cows [32,33,34,35,36].

#### 4.2.1. Glutathione Metabolism

Glutathione (GSH) is a major compound in mammalian cells and has the ability to protect cells from oxidative damage and maintain cell survival and proliferation under stress conditions [37]. In this study, the greater intracellular concentration of GSH in BMECs incubated at 42 °C was consistent with a previous study that demonstrated increased GSH activity in dairy cows under heat stress [38]. Moreover, GSH was confirmed as a tripeptide comprising glycine (Gly), cysteine (Cys) and glutamate (Glu), suggesting a greater requirement for the three amino acids of BMECs under heat stress [39]. This is consistent with the greater Glu concentration determined in the BMECs based on VIP analysis. Therefore, we speculate that BMECs have the ability to resist HS by enhancing the utilization of Glu and by GSH synthesis.

#### 4.2.2. Phenylalanine, Tyrosine and Tryptophan Metabolism

Phenylalanine (Phe) was confirmed as a potentially limiting amino acid for milk protein synthesis in BMECs [40]. The extraction of Phe by the mammary gland was shown to be equal to its amount secreted in milk [18]. With the catalysis of Phe hydroxylase, Phe could be metabolized to tyrosine (Tyr). In this study, greater intracellular concentrations of Phe and Tyr were determined in the HS group relative to that in the control group. However, the lower mRNA expression of SLC7A5, which is an amino acid transporter with high affinity for Phe and Tyr, was also observed in the HS group [41]. Therefore, it seems plausible that HS might inhibit the utilization of Phe for milk protein synthesis but enhance the metabolized action of Phe to Tyr in BMECs. Moreover, Tyr was demonstrated to be able to mitigate the damage of HS as the precursor of the catecholamine neurotransmitters, dopamine and norepinephrine [42]. We speculated that the action metabolizing Phe to Tyr is a self-protective mechanism to resist HS in BMECs.

Tryptophan (Trp) is recognized to play an important role in the metabolism, development and growth of animals [43]. In addition, it is also an essential amino acid for milk protein synthesis. Trp is also a precursor to active molecules such as melatonin, a metabolite that has antioxidative effects [41]. It was demonstrated that the supplementation of rumen-protected Trp increased milk yield and milk protein production in dairy cows during HS [44]. Given that there is no influence of Trp deletion on milk protein yield [45], it is reasonable to speculate that the greater intracellular Trp concentration and activity of the melanogenesis pathway is beneficial to BMECs during HS.

#### 4.2.3. Valine, Leucine and Isoleucine Metabolism

Branched-chain amino acids (BCAAs) are among the essential amino acids with a high concentration (>50%) in the milk protein of dairy cows, and the extraction of BCAAs exceeding the amount of BCAAs secreted in milk [46]. BCAAs not only act as building blocks for milk protein synthesis but also possess other metabolic functions, which were catabolized extensively in lactating mammary tissue to provide amino groups for the biosynthesis of other amino acids [47]. In this study, the intracellular concentrations of valine (Val), leucine (Leu) and isoleucine (Ile) in the HS group were greater relative to the control group, indicating that BMECs have higher BCAA demands under HS. Furthermore, BCAAs have also been confirmed as precursors for signaling molecules [48]. Additionally, it was demonstrated that BCAAs could promote protein synthesis rates in bovine mammary cells by activating the mTOR pathway [49,50]. Given the greater mRNA expression of genes of the mTOR signaling pathway and *CSN1S2*, we speculated that higher levels of intracellular BCAAs were important to milk protein synthesis regulation in BMECs under HS.

#### 4.2.4. Arginine and Proline Metabolism

Arginine (Arg) is recognized as a semi-essential amino acid, with the character of extensive catabolism to other metabolites in mammals [51]. However, Arg has been confirmed as an essential amino acid (EAA) for dairy cows, with a higher expression of arginase occurring in the mammary gland [52]. The higher concentration of citrulline but lower concentration of fumarate, both upstream metabolites of Arg synthesis, indicated the extensive catabolism of Arg in BMECs under HS. Furthermore, our study showed that the downstream metabolites (Glu, Pro, Orn and creatine) of Arg catabolism had a greater concentration in the HS group compared with that in the control group. This increase suggests that HS promotes the activity of the Arg–ornithine–Pro metabolism pathway; HS might increase the activity of ornithine aminotransferase to generate Δ1-l-pyrroline-5-carboxylate (P5C) [53]. Conversely, the lower concentration of putrescine, spermidine and spermine in HS suggested inhibited ornithine–putrescine–spermine metabolism via the decreased the activity of spermidine synthase and spermine synthase [53]. Creatine, another metabolite, was observed at a higher concentration in the HS group. Given that proline, spermine and creatine were not converted to Arg as the end metabolism products of Arg, it is reasonable to speculate that Arg catabolism plays an important role in resisting HS in BMECs. However, further study must understand the regulated mechanism of HS relative to Arg metabolites in BMECs. Additionally, Arg addition has a positive effect on milk protein synthesis regulation through activation of the mTOR signaling pathway in bovine mammary glands under HS [19]. Thus, the greater intracellular Arg concentration observed in the HS group of our study suggests that an increasing Arg requirement of BMECs contributes to the cells’ anti-HS mechanisms.

#### 4.2.5. Alanine, Aspartate and Glutamate Metabolism

Transamination plays an important role in initiating the degradation of alanine (Ala), aspartate (Asp) and Glu to yield pyruvate, oxaloacetate and alpha ketoglutarate (α-KG), respectively, which may provide a carbon source for the tricarboxylic acid cycle (TCA) cycle and adenosine triphosphate (ATP) for the synthesis of purine and pyrimidine nucleotides [54,55]. In this study, HS increased the concentration of glutamine, pyruvic acid, citrate and aspartic acid and reduced the concentration of alanine, indicating that HS promotes the transamination of Ala, Asp and Glu to provide a carbon source for the TCA cycle. Meijer et al. concluded that alanine signaling could adjust glycolysis and gluconeogenesis to maintain glucose synthesis throughout a period of feed-restriction stress [56]. Additionally, Glu, located in the center of ammonia–nitrogen exchange, is a major vehicle for most non-essential amino acids, such as ornithine, citrulline, Pro and Arg, and is an essential precursor for the synthesis of molecules, including nucleotides, amino sugars, and nicotinamide adenosine dinucleotide (phosphate) (NAD(P)). Upregulated glutaminolysis can compensate for the loss of αKG, and its replenishment into the TCA cycle maintains ATP and GSH levels under oxidative stress [57,58]. Therefore, the notably increased level of Glu in our study might suggest an interference in the TCA cycle, influencing energy metabolism, which to a certain extent reflected the HS effect in MAC-T BMECs.

#### 4.2.6. Cysteine and Methionine Metabolism

Methionine (Met) is an important restrictive amino acid for dairy cows, and its deficiency reduces the availability of other essential amino acids [18]. The extraction of Met was shown to be equal to its amount secreted in milk [41]. Met and Cys could be used as precursors of S-adenosylmethionine (SAM), taurine, hydrogen sulfide and glutathione [59]. In this study, HS increased the concentration of Met and SAM, indicating that HS might promote Met catabolism and inhibit the utilization of Met for milk protein synthesis. In addition, the concentrations of SAM and 5’-methylthioadenosine, the principal donors of methyl groups, were increased, showing that HS promoted the one-carbon metabolism of Met and its metabolization to homocysteine, followed by its rapid conversion to cystathionine and then to taurine and glutathione via the transsulfuration pathway, to alleviate oxidant stress induced by various oxidants and protect the tissue from damage [60,61].

**Figure 7:**
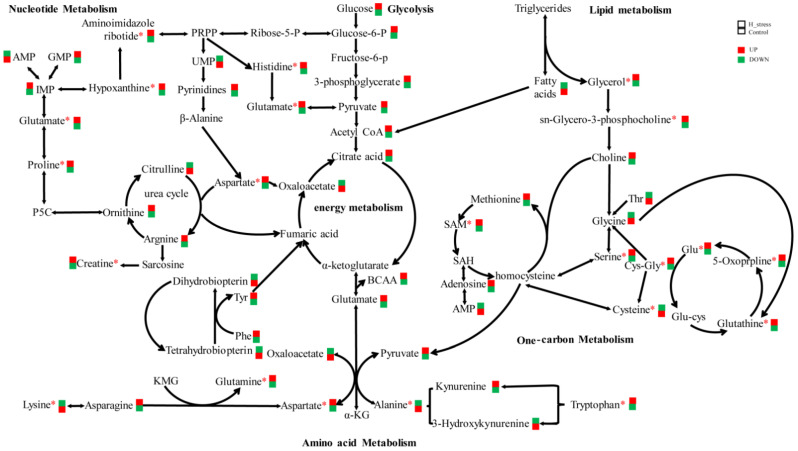
Intracellular metabolic network in the MAC-T bovine mammary epithelial cell line cultured at 42 °C for 12 h. The red boxes represents the higher concentration of the molecule and the green boxes represents the lower one. * *p* < 0.05.

In the present study, HS had the most significant effect on intracellular amino acid metabolism in MAC-T and led to greater intracellular concentrations of some amino acids (Leu, Ile, Val, Phe, Tyr, Pro, Arg, Met, Trp, Glu, Asp and GSH) and decreased the concentrations of other amino acids (Thr, Lys, Ala, Gly and Cys) (Figure 7). The metabolites in the MAC-T bovine mammary epithelial cell exposure to HS might be used as a protein precursor, or as a methyl donor, or as an antioxidant, or act as a signal molecule, to provided ample substrates and energy available for milk protein synthesis supplementing into the amino acid metabolism, energy metabolism and one-carbon metabolism. Metabolites related to amino acid metabolism are shown in Supplemental Table S3.

### 4.3. Amino Acid Transporters

The metabolomics analysis based on LC–MS found changes in intracellular amino acid concentrations and metabolism. Previously, it was demonstrated that amino acids could not diffuse across the cell membrane because of the selective barrier function [17]. Therefore, measuring the change in amino acid transporters is important for the elucidation of the mechanisms involved in the altered concentrations of metabolites that were observed in this study. In our study, HS led to greater mRNA expression of *SLC43A1*, *SLC38A9*, *SLC36A1* and *SLC3A2,* suggesting that HS stimulated the activity of these amino acid transporters, consistent with the results of our metabolomics analysis, which show that HS increased the amino acid intracellular concentrations in MAC-T cells. Interestingly, a lower mRNA expression of *SLC7A5* and *SLC38A2* was observed in our study compared with that in the control group.

*SLC38A2* was confirmed as one of the Na^+^-dependent amino acid transporters with high sensitivity to short-chain neutral amino acids, including Ser, Gly, Ala and Glu [62]. To our knowledge, few studies have reported the effect of HS on *SLC38A2* in BMECs. A similar decrease in the gene expression of *SLC38A2* induced by HS in the breast muscle of broilers was observed [35]. Moreover, the lower expression of *SLC38A2* was observed to decrease glutamicacid (Gln) consumption and inhibit cell growth [63]. Combined with the observation that HS led to higher Gln and Glu intracellular concentration in the HS group in the present work, it is plausible that the decrease in the gene expression of *SLC38A2* might be one of the regulated ways for reducing the Glu and Gln consumption to adapt to HS in BMECs. Moreover, it was demonstrated that *SLC38A2* functions to regulate the activity of the mTOR and general control nonderepressible 2 (GCN2) pathways related to milk protein synthesis [62]. Therefore, the lower mRNA expression of *SLC38A2* with the higher intracellular concentration of Ser, Ala and Glu in the present study may be characteristic of HS adaptation in BMECs.

*SLC7A5* is a heteromeric amino acid transporter (HAT), alongside *SLC3A2* [64]. HATs have been demonstrated to be responsible for the transportation of various amino acids, e.g., essential amino acids (Leu, Ile, Val, Phe, Met, His, Trp, Thr, Arg and Lys). The *SLC7A5* was confirmed to possess an amino acid transportation function, e.g., as an HAT [65]. HS led to a greater intracellular concentration of most essential amino acids (Leu, Ile, Val, Phe, Met, Trp and Arg) based on the metabolomics analysis in this study. Our previous study found that an increase in Thr, Ile and Val decreased the gene expression of *SLC7A5* [17]. Therefore, the lower mRNA expression of *SLC7A5* suggests that a potential negative feedback mechanism related to *SLC7A5* was activated for the regulation of intracellular essential amino acid concentrations to adapt to HS in BMECs. Moreover, the lower gene expression of *SLC7A5* was observed to promote cell apoptosis and cell cycle arrest [66,67]. *SLC7A5* is a potentially important target to study the HS adaptions of BMECs.

Overall, our data in the present study indicate that BMECs could satisfy the amino acid requirement during HS partly by regulating the activity of amino acid transporters. However, amino acid transporters not only play a role in the response to intracellular amino acid concentration but also serve as the signaling molecules to regulate the mTOR pathway activity and milk protein synthesis [68,69]. It is necessary to further determine the transcription level of mTOR pathway activity and milk protein synthesis.

### 4.4. mTOR Signaling Pathway

The mTOR pathway is a key nutrient-sensing pathway that has been well studied [70]. The mammalian target of rapamycin is a protein kinase and serves as the central regulator responsible for integrating various cellular signaling cascades, especially those derived from amino acids [68]. A previous study similarly observed that HS could promote the gene or protein expression of mTOR [71]. Additionally, it was demonstrated that essential amino acids (Leu, Ile, Thr, Met, Arg, Trp and Lys) and non-essential amino acids (Glu and Gln) have the ability to improve the expression of mTOR [72,73]. It is plausible that BMECs could regulate the gene expression of mTOR, partly through intracellular amino acid metabolism and concentration to adapt to HS.

*AKT*, *TSC1*, *TSC2* and *RHEB* are the key upstream signaling molecules that regulate the activity of the mTOR pathway, and *EIF4EBP1*, *eIF4E*, *eEF2K* and *eEF2* are the important downstream regulators of the mTOR pathway [74]. For the upstream activity of mTOR, *AKT* and *RHEB* are the positive regulators of mTOR, and *TSC1* and *TSC2* are the negative regulators of mTOR [75]. The downstream regulators of *eIF4E* and *eEF2* were demonstrated with the function of promoting mTOR pathway activity. *EIF4EBP1* and *eEF2K* were confirmed as mTOR pathway inhibitors [76]. Therefore, our finding of the greater mRNA expression of *AKT*, *RHEB* and *eIF4E* and the lower mRNA expression of *TSC1* and *TSC2* further suggests that BMECs could stimulate mTOR pathway activity for HS adaption. Due to the fact that eEF2K/eEF2 mediates the ribosome translocation through activating *eEF2K* to inhibit *eEF2*, the greater expression of *eEF2K* and lower expression of *eEF2* in the present work indicated that HS could the inhibit the translocation of ribosomes to influence milk protein synthesis in BMECs. Overall, our data related to the gene expression of the mTOR pathway in the present work suggested that BMECs could regulated mTOR pathway activity, partly by sensing the change in intracellular amino acid concentration, to adapt to HS.

### 4.5. Milk Protein Synthesis

The mRNA expression of casein is positively correlated with milk yield, and MAC-T cells have been widely used to study milk protein synthesis regulation based on the transcription level [17]. In the present work, the abundance of the casein gene was used to evaluate the potential effects of HS on milk protein synthesis. The greater *CSN1S2* expression is consistent with the increase in amino acid intracellular concentration and metabolism, and the stimulation of mTOR pathway activity further suggesting that BMECs possess the potential ability to protect from HS and partly maintain or recover the capacity for αs2-casein protein synthesis. However, there was no significant change in the expression of *CSN1S1* and *CSN2*, which comprise more than 65% of milk proteins [77]. Previous studies have also shown that there was no significant change in the milk protein yield of dairy cows during HS [9]. Thus, further study should be performed to elucidate the regulated mechanisms related to different milk protein components in HS.

## 5. Conclusions

In the present study, our data provided more systematic evidence based on metabolomics and gene expression that BMECs might possess the ability to resist HS damage and continue milk protein synthesis partly by enhancing intracellular amino acid absorption and metabolism and activating the mTOR signaling pathway. Considering that the concentrations of most of the intracellular essential amino acids were increased, future studies might focus on the essential amino acid requirements of bovine mammary glands in HS.

## Data Availability

All data presented in this study are available on request from the corresponding authors.

## Supplementary Materials

The following are available online at https://www.mdpi.com/article/10.3390/ani11113153/s1, Table S1: GenBank accession number, hybridization position, sequence, amplicon size of primers used, Table S2: The results of identifiable metabolites, Table S3: The concentration of metabolites in the intracellular metabolic network in MAC-T cells.
