# Supplementary material for: Effect of Heat Stress on Bovine Mammary Cellular Metabolites and Gene Transcription Related to Amino Acid Metabolism, Amino Acid Transportation and Mammalian Target of Rapamycin (mTOR) Signaling
Source: Animals (Basel). 2021 Nov 4;11(11):3153. doi: 10.3390/ani11113153 (PMC8614368; doi:10.3390/ani11113153)
Supplement: Supplementary file 1 [file animals-11-03153-s001.zip › animals-1364526-supplementary.pdf]

Supplementary Information

Table S1. Gene name, GenBank accession number, Forward and reverse primer sequence

Gene	Accession#	Forward primer	Reverse Primer
HSF-1	XM_005215151	GCCATGAAGCACGAGAACGA	GTTTGACTGCACCAGCGAGATG
HSPB8	NM_001014955	GGAGGTGTCTGGTAAACACGAAG	GCTCTCTCCAAACGGTGAGTAA
HSPA5	XM_024998380	GATCAAGGCAACCGCATCAC	GCTGCACGGACGGGTCATT
HSP90AB1	NM_001079637	GCTCAGACGAGGAGGATGATAGT	CCAAGTGATCTTCCCAGTCATT
HSPA1A	NM_203322	ACGACGGAGACAAGCCTAAG	GTCAGCACCATCGACGAGA
CSN1S1	XM_005208027	CCCAACAGAAAGAACCTATG	CCAATGGGATTAGGGATG
CSN1S2	XM_024993018	AATCAGCCAGCGTTACCAG	CCAGTCCAACCATAACCAGG
CSN2	XM_010806178	GTGAGGAACAGCAGCAAACA	CCAGGAGCAAAACCAAGAAC
SLC3A2	NM_001024488	GAGCATTCCCTTGCTTGCAC	GCTCATGGTGCCTGAGTCG
SLC7A5	NM_174613	CCGTACCCTCACTGGTGTTC	AGATGAACCTTGATGGGCCG
SLC36A1	XM_015472327	AGGGTCCTACATGCGGTTTG	GGGGACCCATCAAGATGCC
SLC38A2	XM_024991403	TGAAAAGCCATTATGCCGATGT	CCCACAATCGCATTGCTCAG
SLC38A9	NM_001102163	GGGCATAAAACAGGCTGGGT	CTTGACCACTGCCCAAAGGA
SLC43A1	NM_001206598	TGGTACCCGTCCAAACACTT	AGGAACCCCAGGAGTGAGAA
AKT	NM_173986.2	CTGCACAAGCGAGGTGAGTA	GAGAAGTTGTTGAGGGGCGA
TSC1	XM_010819002.2	TACTGGGCCACGTCGTGAG	CGTCGGTGTCCATCTTGAGAC
TSC2	XM_015469258.1	GCAGCAGGATCCAGACCTCT	GTCTCTGTGAGCTCCAGGTGG
RHEB	NM_001031764	GCTAAGATGCCGCAGTCCA	CGTCAACGAGGATTTCCCC
mTOR	NM_001144096.3	CTTCTTCCGTTCCATCTC	CTTCCACTAAGGCTTCAT T
EIF4EBP1	NM_001077893	GGAGTGTCGGAACTCACCTG	AACTGTGACTCTTCACCGCC
eIF4E	NM_174310.3	AGGGAGGGTATACAAGGAAAGGTT	TTTTAGTGGTGGAGCCGCTC
eEF2K	NM_175813	TCTCTGTCCTCAATCAAG	GGTCTCATCTGTATCTGT
eEF2	NM_001075121	GAGATCCAGTGTCCAGAA	GAAGCCAAAGGACTCATT
RPS9	XM_001101152	CCTCGACCAAGAGCTGAAG	CCTCCAGACCTCACGTTTGTTC
GAPDH	XM_001034034	TGGAAAGGCCATCACCATCT	CCCACTTGATGTTGGCAG
UXT	XM_001037471	TGTGGCCCTTGGATATGGTT	GGTTGTCGCTGAGCTCTGTG

Note: Heat shock factor 1, HSF-1; Heat shock protein beta-8, HSPB8; Heat shock protein 5, HSPA5; Hheat shock protein 90 kDa alpha class B member 1, Hsp90AB1; Heat shock 70 kDa protein 1A, HspA1A; αS1-casein, CSN1S1; αS2-casein, CSN1S2; β-casein, CSN2; 4F2 cell-surface antigen heavy chain, SLC3A2; Large neutral amino acids transporter small subunit 1, SLC7A5; Proton-coupled amino acid transporter 1, SLC36A1; Sodium-coupled neutral amino acid transporter 2, SLC38A2; Large neutral amino acids transporter small subunit 3, SLC43A1; Sodium-coupled neutral amino acid transporter 9, SLC38A9; Serine/threonine-protein kinase, AKT; Tuberous sclerosis complex 1, TSC1; Tuberous sclerosis complex 2, TSC2; GTP-binding protein Rheb, RHEB; Mammalian Target of Rapamycin, mTOR; Eukaryotic translation initiation factor 4E binding protein 1, EIF4EBP1; Eukaryotic translation initiation factor 4E, eIF4E; Eukaryotic elongation factor 2 kinase, eEF2K; Elongation factor 2, eEF2; 40S ribosomal protein S9, RPS9; Glyceraldehyde-3-phosphate dehydrogenase, GAPDH; Ubiquitously expressed transcript protein, UXT.

Table S2. The results of identifiable metabolites

type	All	MS2	MS1	known(MS1+MS2)	unknown
POS	12176	499	6668	7167	5009
NEG	9737	197	5823	6020	3717

Table S3. The concentration of metabolites in the intracellular metabolic network in MAC-T cell

Metabolites	Pvalue	fdr	vip	UP/DOWN
Oleamide	0.4223	0.7039	18.1061	DOWN
Glutathione	0.0003	0.0143	17.2101	UP
Erucamide	0.0029	0.0476	13.6265	UP
3-Guanidinopropanoate	0.0001	0.0055	13.2997	UP
Allopurinol	0.0024	0.0430	12.8180	UP
Phenylalanine	0.0544	0.2783	7.6050	UP
Hypoxanthine	0.0168	0.1443	7.2583	UP
Inosine	0.0059	0.0740	6.8730	UP
L-Norleucine	0.0043	0.0617	6.7312	DOWN
Oleoyl Ethyl Amide	0.5217	0.7656	6.4756	DOWN
Stearamide	0.4155	0.7000	5.9004	UP
Isoleucine	0.0347	0.2221	5.7392	UP
Indoline	0.0653	0.3065	5.5393	UP
Tyrosine	0.1403	0.4600	5.3867	UP
Pyroglutamic acid	0.0109	0.1084	5.2751	UP
Epigallocatechin	0.0000	0.0005	4.5760	DOWN
Palmitic amide	0.5633	0.7895	4.4359	DOWN
5'-Methylthioadenosine	0.0529	0.2736	4.3901	UP
Tryptophan	0.0281	0.1960	4.2527	UP
Glutamate	0.0004	0.0147	4.2018	UP
Proline	0.0478	0.2596	4.1041	UP
Stearic acid	0.1227	0.4288	4.1035	DOWN
Guanine	0.0042	0.0604	4.0617	UP
L-threo-Sphingosine C-18	0.0005	0.0171	3.9782	UP
Niacinamide	0.0324	0.2125	3.6737	UP
Deoxyguanosine 5'-monophosphate	0.0002	0.0108	3.5411	UP
2-Amino-2-methylbutanoate	0.2926	0.6133	3.2540	UP
N1-Acetylspermidine	0.0003	0.0138	3.2486	UP
Dihomo-gamma-Linolenic Acid	0.0425	0.2440	3.1404	DOWN
Arachidonic Acid	0.0348	0.2223	3.0759	DOWN
D-Glycerate 2-phosphate	0.0000	0.0005	3.0121	DOWN
Piperidine	0.0332	0.2151	2.9379	UP
Pantothenic acid	0.0033	0.0520	2.8644	UP
sn-Glycero-3-phosphocholine	0.0165	0.1431	2.7575	UP
Guanosine	0.1189	0.4218	2.6041	UP
Cys-Gly	0.0008	0.0208	2.5300	DOWN
5-oxo-D-proline	0.0000	0.0044	2.4762	UP
Palmitoleic acid	0.2115	0.5403	2.1782	DOWN
S-Adenosyl-methionine	0.0082	0.0925	2.0626	UP
Adenosine	0.1124	0.4103	2.0509	UP
Choline	0.2834	0.6051	2.0471	UP
Methionine	0.0619	0.2985	2.0343	UP
Histidine	0.0336	0.2168	1.9398	UP

Glutamine	0.0286	0.1978	1.7607	UP
a-Linolenic acid	0.2500	0.5743	1.5789	DOWN
Aspartate	0.0001	0.0054	1.4946	UP
Valnine	0.3863	0.6799	1.3183	UP
Dihydrobiopterin	0.0596	0.2921	1.1932	DOWN
Aminoimidazole ribotide	0.0036	0.0543	1.1866	UP
Argnine	0.2437	0.5685	1.1569	UP
Uridine diphosphate glucuronic acid	0.0051	0.0686	1.1235	UP
Putrescine	0.0891	0.3638	1.1212	DOWN
3-phosphoglycerate	0.0000	0.0034	1.1035	UP
D-3-phosphoglycerate	0.0000	0.0034	1.1035	UP
Adrenic Acid	0.2978	0.6183	1.0834	DOWN
Spermidine	0.3198	0.6357	1.0267	DOWN
Cytosine	0.0896	0.3653	0.8995	UP
Taurine	0.3427	0.6524	0.8466	UP
Lysine	0.0475	0.2593	0.7808	DOWN
Alanine	0.0043	0.0612	0.7641	UP
Serine	0.0223	0.1717	0.7332	UP
Glycerol	0.0033	0.0513	0.6569	UP
Creatine	0.0001	0.0095	0.6244	UP
Uracil	0.2443	0.5687	0.6097	UP
Nervonic acid	0.2904	0.6108	0.6078	DOWN
Citrulline	0.3782	0.6746	0.5923	DOWN
Caprylic acid	0.0154	0.1358	0.5649	DOWN
Threonine	0.3446	0.6532	0.5589	DOWN
Thiamin diphosphate	0.0002	0.0125	0.5011	DOWN
4-Methyl-2-oxopentanoate	0.0989	0.3852	0.4278	UP
Asparagine	0.0812	0.3456	0.3964	UP
(S)-Malate	0.2434	0.5682	0.3841	UP
3-Hydroxykynurenine	0.2290	0.5566	0.3680	DOWN
Myristic acid	0.0190	0.1549	0.3436	DOWN
Cysteine	0.0377	0.2307	0.3296	UP
Kynurenine	0.1068	0.3994	0.2267	UP
Ornithine	0.6958	0.8618	0.1512	UP
UMP	0.2150	0.5440	0.1507	DOWN
Fumarate	0.3808	0.6763	0.1259	DOWN
GMP	0.5978	0.8090	0.1104	UP
Acetyl CoA	0.0231	0.1747	0.0947	UP
Glucose	0.4437	0.7207	0.0880	UP
Glycine	0.4262	0.7074	0.0874	DOWN
Tetrahydrobiopterin	0.6236	0.8203	0.0814	DOWN
1-Pyrroline-2-carboxylate	0.6191	0.8196	0.0738	UP
Leucine	0.7887	0.9119	0.0476	UP
Pyruvate	0.9543	0.9837	0.0464	DOWN

Glucose-6-P	0.4605	0.7301	0.0451	UP
Citrate acid	0.8106	0.9227	0.0376	UP
Hydroxypyruvate	0.8939	0.9608	0.0289	DOWN
Spermine	0.6321	0.8246	0.0216	DOWN
Cysteinyl glycine	0.7045	0.8658	0.0111	UP
D-glycerate	0.8430	0.9384	0.0091	DOWN
AMP	0.8418	0.9384	0.0090	DOWN
Oxaloacetate	0.8384	0.9365	0.0064	DOWN
